#  Reconstruction by Pancreaticogastrostomy versus Pancreaticojejunostomy following Pancreaticoduodenectomy: A Meta-Analysis of Randomized Controlled Trials

**DOI:** 10.1155/2012/627095

**Published:** 2012-02-19

**Authors:** YinFeng Shen, WenYin Jin

**Affiliations:** Surgery, Chinese Medicine Hospital of Hubei Province, Hubei University of Chinese Medicine, Wuhan 430061, China

## Abstract

*Objectives*. The aim of our study was to evaluate and compare the results of pancreaticogastrostomy (PG) and pancreaticojejunostomy (PJ) after pancreaticoduodenectomy (PD). *Methods*. Published data of randomized clinical trials (RCTs) comparing the clinically relevant outcomes of PG versus PJ after PD were analyzed. Two reviewers assessed the quality of each trial and collected data independently. The Cochrane Collaboration's RevMan 5.0 software was used for statistical analysis. Proportions were combined, and the odds ratio (OR) with its 95% CI was used as the effect size estimate. *Results*. Four RCTs published in 1995 or later were included in this meta-analysis, in which 276 patients underwent PG and 277 patients underwent PJ followed PD. In the combined results of PG versus PJ, a significant difference in the morbidity of intra-abdominal complications (OR, 0.34; 95% CI, 0.23–0.49; *P* < 0.00001) was found, but no significant difference could be found for pancreatic fistula (OR, 0.69; 95% CI, 0.42–1.12 , *P* = 0.13) mortality (OR, 1.09; 95% CI, 0.42–2.83; *P* = 0.87), recovery with no complications (OR, 1.26; 95% CI, 0.90–1.78; *P* = 0.18), biliary fistula (OR, 0.55; 95% CI, 0.22–1.35; *P* = 0.19), or in delayed gastric emptying (OR, 0.55; 95% CI, 0.33–1.01; *P* = 0.06). *Conclusions*. Current RCTs suggest that PG is better than PJ for pancreatic reconstruction after PD.

## 1. Introduction

With dramatic improvement in operative mortality, pancreaticoduodenectomy (PD) has become increasingly accepted as a safe and appropriate operation for selected patients with periampullary tumors, pancreatic head cancer, benign neoplasms, and other non-neoplastic conditions such as chronic pancreatitis [1]. With advances in treatment techniques, the mortality rate of PD has decreased to below 5% in many institutions around the world in recent years [1–5]. However, even with these advancements in operative technique and postoperative management, postoperative morbidity of intra-abdominal complications remains high even in large series [4]. The most common complications after PD are pancreatic fistula, delayed gastric emptying, biliary fistula, and wound infection [6–8]. They often contribute significantly to prolonged hospitalization and mortality [6]. Leakage from the pancreatic anastomosis remains the single most important cause of morbidity and sometimes mortality [1].

Recently, considerable attention has been focused on refinements in operative technique for PD, especially on the management of the pancreatic remnant, with the intent to decrease the incidence of pancreatic fistula. These efforts include technical modifications such as the pancreatojejunal anastomosis technique, the pancreatogastric anastomosis, and external drainage of the pancreatic duct [5]. Pancreaticogastrostomy (PG) and pancreaticojejunostomy (PJ) have been the most commonly used method of restoring pancreatioenteric continuity after PD. Some retrospective studies [9–11] and one RCT [12] have reported lower pancreatic fistula rate with PG instead of PJ, and a recent meta-analysis [13] suggested that the safer means of pancreatic reconstruction after PD was PG. However, 3 RCTs [14–16] showed PG and PJ to be similar in regards to pancreatic fistula rates, and a recent meta-analysis concluded [17] that PG and PJ were not different in terms of pancreatic fistula rate or overall morbidity rate.

Thus, in order to establish which is the best technique for pancreatoenteric anastomosis, it is important to identify the definition of pancreatic fistula used, before any series of patients can be compared [1]. To evaluate and compare the results of PG and PJ after PD, we performed an up-to-date meta-analysis to PG versus PJ including all RCTs, and when appropriate and possible, to establish the sources of heterogeneity in the results.

## 2. Materials and Methods

### 2.1. Data Sources

We performed a systematic review of the literature published between 1990 and July 2011. To identify studies published from 1990 to July 2011, we performed a comprehensive search of abstracts in the MEDLINE database, OVID database, Springer database, the Science Citation Index, and the Cochrane Library database with use of the following search terms: “pancreaticoduodenectomy,” “pancreaticogastrostomy,” “pancreaticojejunostomy,” with limitations to Randomized Controlled Trial, Humans. Reports in any language were eligible for inclusion. To avoid double counting, two data extractors compared the articles for participating institutions and inclusion criteria. Unpublished research was not included.

### 2.2. Inclusion and Exclusion Criteria

Only RCTs were included. Any etiology for PD was eligible, and there was no limitation because of race, gender, or age. Comparator intervention was considered PG, while control intervention was considered PJ.

### 2.3. Statistical Analysis

Two independent reviewers extracted data by using a specially developed form and entered it into the freeware program Review Manager (Version 5.0 for Windows, Cochrane Collaboration, Oxford, UK, 2008), respectively. The odds ratio (OR) for each trial was calculated from the number of evaluable patients, and ORs with their two-sided 95% CIs were used for dichotomous outcomes as the confirmatory effect size estimate and test criterion. For continuous variables, weighted mean difference (WMD) was calculated with 95% confidence intervals. In the course of data combination, heterogeneity was evaluated with the Cochran Q test. The fixed-effects model and random-effects model were applied. The hypothesis tests were based on the 95% CIs, and *P* values were used for illustration. All *P* values were two-sided, and *P* < 0.05 was considered statistically significant. To determine the potential risk bias in the overall results from the inclusion of studies that violated some of the eligibility criteria, sensitivity analysis and publication bias analysis were performed.

## 3. Results

### 3.1. Trial and Patient Characteristics

**Figure 1:**
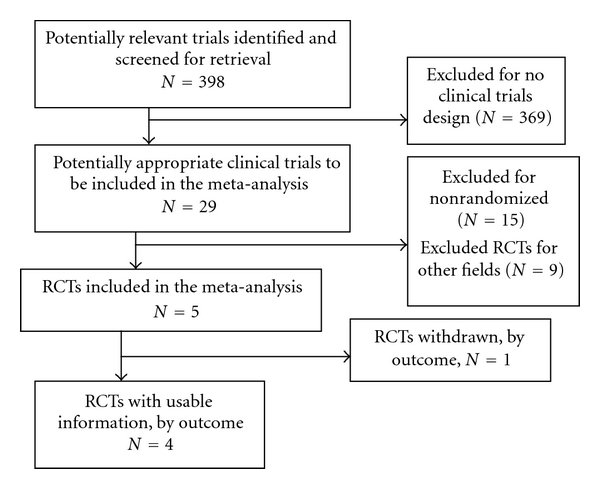
QUOROM flow diagram of included and excluded studies.

A total of 398 studies were retrieved, and the process of identifying relevant trials is shown in Figure 1. Among these 398 studies, 369 were excluded because of trial design, 29 studies were potentially appropriate clinical trials to be included in the meta-analysis, 15 were excluded because of absence of randomization, and 9 were excluded RCTs for other reasons. Finally, five RCTs were included [12, 14–16], which were all published as full articles; clinically relevant outcomes for our study could not be extracted from one of these five, thus leaving four RCTs for meta-analysis. Among these 4 studies, there were a total of 276 patients that underwent PJ and 277 patients that underwent PJ. The main characteristics of the four included studies are reported in Table 1.

### 3.2. Results of Meta-Analysis

#### 3.2.1. Morbidity of IACs

**Figure 2:**
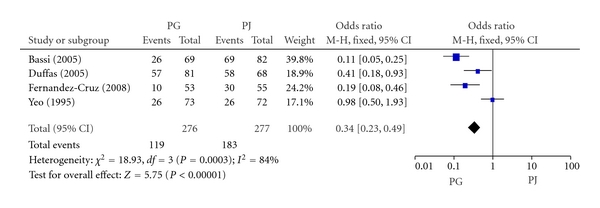
Forest plot of morbidity of IACs between PG and PJ.

The intra-abdominal complications (IACs) included pancreatic, biliary, or digestive tract fistula, intra-abdominal collections (either infected [abscess] or not), acute pancreatitis, cholangitis; intra-abdominal or digestive tract hemorrhage, delayed gastric emptying, and wound disruption (either infected or not). The four included RCTs involved 553 patients reported IACs. The morbidity of IACs in PG group and PJ group was 43.1% (119/276) and 66.1% (183/277), respectively. Meta-analysis showed a significant difference in morbidity of IACs between PG group and PJ group (OR, 0.34; 95% CI, 0.23–0.49; *P* < 0.00001) (Figure 2).

#### 3.2.2. Pancreatic Fistula

**Figure 3:**
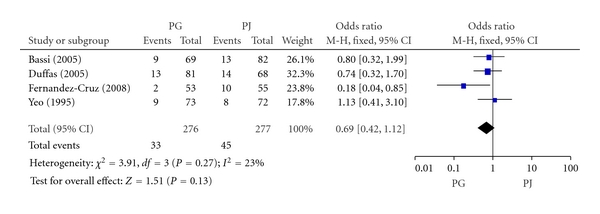
Forest plot of pancreatic fistula between PG and PJ.

The included RCTs reported on pancreatic fistula. The rate of pancreatic fistula in the PG group and PJ group was 12.0% (33/276) and 16.3% (45/277), respectively. Meta-analysis showed no significant difference in pancreatic fistula between PG and PJ group (OR, 0.69; 95% CI, 0.42–1.12; *P* = 0.13) (Figure 3).

#### 3.2.3. Mortality

**Figure 4:**
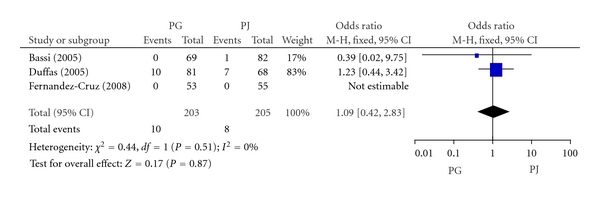
Forest plot of mortality between PG and PJ.

Three included RCTs involving 408 patients reported the mortality. The mortality of PG group and PJ group was 4.9% (10/203) and 3.9% (8/205), respectively. Meta-analysis showed no significant difference in mortality between PG and PJ group (OR, 1.09; 95% CI, 0.42–2.83; *P* = 0.87) (Figure 4).

#### 3.2.4. Recovery with No Complications

**Figure 5:**
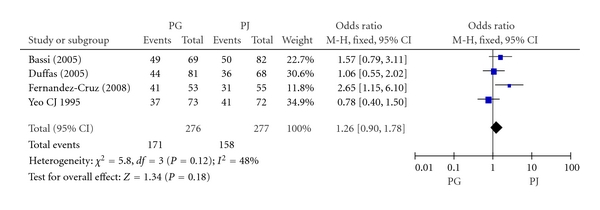
Forest plot of recovery with no complications between PG and PJ.

Four included RCTs including 553 patients reported recovery with no complications. The rate of recovery with no complications in PG group and PJ group was 62.0% (171/276) and 57.0% (158/277) respectively. Meta-analysis showed no significant difference in pancreatic fistula between PG and PJ group (OR, 1.26; 95% CI, 0.90–1.78; *P* = 0.18) (Figure 5). 

#### 3.2.5. Biliary Fistula

**Figure 6:**
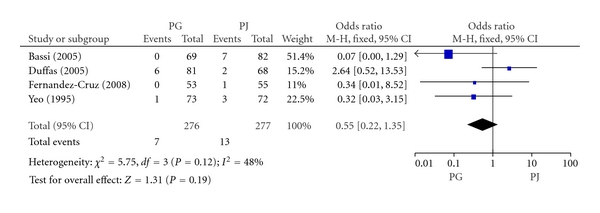
Forest plot of biliary fistula between PG and PJ.

Biliary fistula was defined as bile in the drain fluid from the subhepatic drain (or an operatively placed drain or a subsequently placed percutaneous drain) with the level of total bilirubin exceeding the upper limit of normal. 4 included RCTs including 553 patients reported biliary fistula. The rate of biliary fistula in PG group and PJ group was 2.5% (7/276) and 4.7% (13/277), respectively. Meta-analysis showed no significant difference in biliary fistula between PG and PJ group (OR, 0.55; 95% CI, 0.22–1.35; *P* = 0.19) (Figure 6). 

#### 3.2.6. Delayed Gastric Emptying

**Figure 7:**
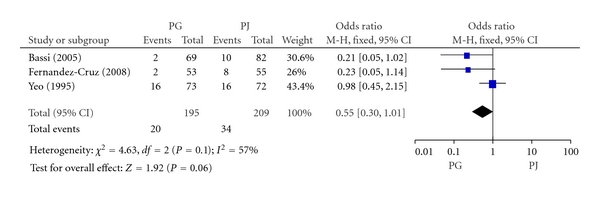
Forest plot of delayed gastric emptying between PG and PJ.

Delayed gastric emptying (DGE) was defined when the nasogastric tube was maintained for ten or more days, combined with one or more of the following: vomiting after removal of nasogastric tube, reinsertion of nasogastric tube, or failure to progress with oral feeding. Three included RCTs involving 404 patients reported delayed gastric emptying. The rate of delayed gastric emptying in PG group and PJ group was 10.3% (20/195) and 16.3% (34/209), respectively. Meta-analysis showed no significant difference in delayed gastric emptying between PG and PJ group (OR, 0.55; 95% CI, 0.33–1.01; *P* = 0.06) (Figure 7).

### 3.3. Sensitivity Analysis and Publication Bias

Sensitivity analysis and publication bias estimates were performed to determine statistically significant results. For intra-abdominal complications (IACs) between PG group and PJ group, combined ORs were calculated with a fixed-effects model and a random-effects model, and the results were compared. The OR with a fixed-effects model was 0.34 (95% CI, 0.23–0.49; *P* < 0.00001); moreover, because statistically significant data are more likely to be published and the findings of the present review were mostly positive, our meta-analysis was likely influenced very little by publication bias. However, because of the small numbers of randomized controlled trials available, more detailed stratification comparisons could not make, which could have influenced the validity of our study to some extent.

## 4. Discussion

To reduce the incidence of postoperative complications, a variety of techniques [18] as well as pharmacologic prophylactic approaches [19, 20] have been used and evaluated over the years in the management of the pancreatic remnant following PD. Pancreatic anastomosis leakage remains a major cause of postoperative morbidity after PD, and it contributes significantly to operative mortality. Pancreatoenteric anastomotic failure is one of the major causes of morbidity because of delayed gastric emptying, pancreatic fistula, and wound infection; pancreatic fistula can also lead to hemorrhage (intra-abdominal and/or into the digestive tract), leakage (biliary and/or digestive tract), intra-abdominal infection, wound disruption (infected or no), and even death. The most common techniques for reconstruction of pancreatic gastrointestinal continuity after PD involve a pancreatico-enteric anastomosis, usually either PJ or PG. The best technique for pancreatic anastomosis is still a challenge for the pancreatic surgeon. The pancreatojejunal anastomosis is carried out either as an end-to-end anastomosis with invagination of the pancreatic stump into the jejunum or as an end-to-side anastomosis with or without duct-to-mucosa suturing [21]. The pancreatogastric anastomosis is performed to the gastric lumen through either the gastric stump or through an anterior wall gastrostomy (in the case of pylorus-preserving PD).

The present meta-analysis showed that PG is better than PJ for pancreatic reconstruction after PD, because PG has lower morbidity of intra-abdominal complications than PJ (*P* < 0.00001), while the two techniques of anastomosis were not different in terms of pancreatic fistula rate (*P* = 0.13), mortality (*P* = 0.87), recovery with no complications rate (*P* = 0.18), biliary fistula rate (*P* = 0.19), and delayed gastric emptying rate (*P* = 0.06).

The technique of PG has several potential advantages over PJ. First, the PG anastomosis can be performed easily, because the posterior wall of the stomach lies immediately anterior to the mobilized pancreatic remnant and is usually wider than the transected pancreas. Second, with PG, the pancreatic exocrine secretions enter the potentially acidic gastric environment, precluding digestive damage of the pancreatoenteric anastomosis by activated proteolytic enzymes. In contrast with PJ, the activation of pancreatic exocrine secretions can occur more easily in the presence of intestinal enterokinase and bile. Third, PG avoids the long jejunal loop where pancreatobiliary secretions accumulate during the early postoperative period. Fourth, postoperative gastric decompression can provide constant removal of pancreatic and gastric secretions avoiding accumulation and thus tension on the anastomosis. Fifth, PG anastomosis reduces the number of anastomoses in a single loop of retained jejunum, which potentially decreases the likelihood of loop kinking. The decreased morbidity of intra-abdominal complications for PG may be the result of the aforementioned theoretical advantages. Published studies have favored PG over PJ [12, 22] although these studies are limited by their small patient populations.

It is generally accepted that compared to a fibrotic pancreatic remnant, a soft and fragile pancreatic remnant frequently results in a high pancreatic anastomosis leakage rate [23]. There are many factors which can lead to pancreatic anastomosis leakage (pancreatic fistula), including pancreatic factors (pancreatic texture, original pathology, blood supply to the pancreas remnant, pancreatic juice output, pancreatic duct size), patient factors (age, gender, level of preoperative jaundice, comorbid illness), and operative factors (operation time, blood loss, type of anastomosis, stenting of pancreatic duct) [1, 24–27]. Among these factors, the main factors include pancreatic texture [1, 27–29], pancreatic stump blood supply, pancreatic duct size [1, 29], and pancreatic juice output [27, 30]. All RCTs which were included in our study reported diverse factors (pancreatic factors, patient factors, and operation factors) which were different between the PG group and the PJ group. For pancreatic fistula, the present study showed no significant difference in two groups (*P* = 0.13). Although there is heterogeneity between the analyzed RCTs, all RCTs were conducted in specialized centers by highly experienced surgeons, and the surgical care is likely to be similar among studies. Regarding methodological quality, we consider our analyses to be relevant [31].

The results of this meta-analysis are in line with research from McKay et al. [13] and are partly similar with Wente et al. [17]. However, our meta-analysis has some limitations. First, due to the lack of specific information in the original papers, we cannot perform a subgroup analysis according to patient age and the etiology of PD; thus, it is unclear whether the advantage of PG is potentially applicable to all subgroups of patients. Second, the reported technique for PD in each RCT was variable with conventional PD, PPPD, or PD plus extended resection (Figure 1). Different operative procedures could lead to different complications. Third, other factors, such as presenting symptoms, preoperative blood parameters, the presence of comorbid illness, and preoperative biliary drainage, could influence the frequency or type of morbidity. Fourth, the definition for pancreatic fistula also varied between RCT, with only one [14], utilizing the ISGPF criteria [24], which could influence our study. Fifth, this meta-analysis included only 553 patients and 4 RCTs, and a type II error may be possible.

In conclusion, the evidence from this formal meta-analysis suggests that PG is better than PJ for pancreatic reconstruction after PD. PG can provide an adequate reconstruction for pancreaticoenteric continuity following PD. Future large-scale, high-quality, multicenter trials are still required to clarify the issues of PG reconstruction following PD. For future experiment on PD, the question for the management of the pancreatic remnant must be addressed in the future.

## Figures and Tables

**Table 1:** Characteristics of RCTs Included in the study.

Author	Year	Total No.	Setting	AC	Operation
Bassi et al. [15]	2005	151	Single center	Adequate	PPPD or PD
Duffas et al. [16]	2005	149	Multicenter	Adequate	PPPD or PD or ER
Fernàndez-Cruz et al. [12]	2008	108	Single center	Adequate	PPPD
Yeo et al. [14]	1995	145	Single center	Adequate	PPPD or PD

Abbreviation: AC = allocation concealment; PPPD = pylorus-preserving pancreaticoduodenectomy; PD =pancreaticoduodenectomy; ER = extended resection. *PPPD or PD plus resections extended to other organs (colon, small intestines, mesenteric portal confluence, liver, biliary tree).
